# Hyperspectral and machine-learning-based classification of ischemic intestinal tissue

**DOI:** 10.1117/1.JBO.30.11.116001

**Published:** 2025-10-31

**Authors:** Valery V. Shupletsov, Ilya A. Goryunov, Nikita A. Adamenkov, Andrian V. Mamoshin, Elena V. Potapova, Andrey V. Dunaev, Viktor V. Dremin

**Affiliations:** a Orel State University, Research & Development Center of Biomedical Photonics, Orel, Russia; b Orel Regional Clinical Hospital, Orel, Russia; c The National Medical Research Center of Surgery Named After A. Vishnevsky, Moscow, Russia; d Aston University, College of Engineering and Physical Sciences, Birmingham, United Kingdom

**Keywords:** hyperspectral imaging, machine learning, XGBoost, intestinal ischemia, tissue oxygen saturation

## Abstract

**Significance:**

Accurate intraoperative assessment of intestinal tissue viability is critical in determining the extent of resection in cases of intestinal ischemia. Current evaluation methods are largely subjective and lack the precision required for reliable decision-making during surgery.

**Aim:**

We aim to develop and validate a hyperspectral imaging (HSI) system combined with machine learning (ML) to objectively assess intestinal wall viability and differentiate between reversible and irreversible ischemia.

**Approach:**

A portable HSI system was used to acquire spectral data from rat models with induced intestinal ischemia at different time points (1, 6, and 12 h). Tissue oxygen saturation was calculated using a two-wavelength algorithm. Spectral data were classified using an ML pipeline based on principal component analysis (PCA) and the XGBoost algorithm, trained on histologically validated tissue classes.

**Results:**

Tissue saturation decreased with prolonged ischemia (from 66% in healthy tissue to 21% after 12 h). Classification accuracy using PCA features reached 98% for intact tissue, 95% for possibly reversible ischemia, and 97% for irreversible ischemia. Classification maps closely matched tissue saturation distributions and histological findings. Initial clinical testing confirmed the system’s sensitivity to ischemic changes in human subjects, although further training on human data is required for ML application.

**Conclusions:**

HSI combined with ML provides an effective, non-invasive tool for real-time intraoperative assessment of intestinal viability. This approach improves the objectivity of surgical decision-making and may reduce unnecessary resections.

## 1 Introduction

This paper focuses on the development of a hyperspectral imaging system to assess the degree of ischemia of intestinal wall tissue to determine its viability.

According to the clinical guidelines of the World Society of Emergency Surgery, acute mesenteric ischemia is a group of diseases characterized by impaired blood supply to various segments of the intestine, leading to ischemia and secondary inflammatory changes.^1^ Ischemia results in tissue hypoxia, leading to irreversible necrotic changes in all layers of the intestinal wall. Intestinal ischemia can be non-occlusive in nature and of occlusive etiology including mesenteric artery embolism (50%), mesenteric artery thrombosis (20% to 35%), or mesenteric vein thrombosis (5% to 15%). The results of current research show that acute mesenteric ischemia accounts for 0.09% to 0.2% of hospitalizations, but mortality is as high as 80%.^2^

When there is doubt about the viability of the compromised intestinal segment, surgical treatment for these conditions often involves resection of the affected bowel section. This, in turn, can lead to the development of post-resection complications (such as anastomotic leakage, bleeding, formation of acute anastomotic ulcers, and abscess formation) and digestive impairments (short bowel syndrome).

To date, the generally accepted method for intraoperative determination of intestinal viability is the visual Kerthe method based on the determination of mesenteric vessel pulsation, peristaltic contractions, and assessment of the color of the intestinal wall.^3^^,^^4^ However, these signs are subjective in nature because, by visual determination of intestinal color, only a virtually unchanged intestine can be reliably distinguished from an obviously non-viable intestine. Peristalsis may persist for a long time even after necrosis of the mucosal layer and may be absent in the unaltered intestine. In this case, due to the lack of oxygen supply, there may be sharp contractions of the intestine, which can be mistakenly considered peristaltic. Pulsation of the mesenteric vessels may persist in the presence of severe circulatory disturbance directly in the intestinal wall.^5^ An incorrect assessment of the intestinal wall’s condition can lead to a serious complication: perforation of the ischemic bowel segments. This, in turn, can result in peritonitis and a fatal outcome. Conversely, an unjustified resection of significant portions of the small intestine can lead to the development of short bowel syndrome. This condition manifests as diarrhea; steatorrhea; varying degrees of dehydration; alterations in serum levels of potassium, sodium, chloride, and other electrolytes; chronic polyvitamin deficiency; progressive weakness; paresthesia; dermatitis; disturbances in calcium–magnesium metabolism; depletion of fat stores; and protein–energy malnutrition. Thus, determining reversible ischemic changes from irreversible ones in the intestine will allow surgeons to accurately assess the condition of the intestinal wall and, if necessary, make a decision regarding resection. This will help avoid the development of complications associated with necrosis of the intestinal wall in the postoperative period. At the same time, identifying sections of the intestine with reversible ischemia will prevent the unnecessary expansion of the surgical intervention’s scope and the associated surgical trauma.

The modern approach to many surgical interventions requires a wider range of intraoperative diagnostic information about organ blood microcirculation than just the subjective evaluation of visual criteria.^1^ Current intraoperative techniques [ultrasound Doppler ultrasonography, polarographic method, laser Doppler flowmetry and imaging, laser speckle contrast imaging, lateral darkfield microscopy, fluorescence of oxidative metabolism compartments, optical coherence tomography, photoplethysmography, and indocyanine green (ICG) fluorescence imaging], which provide some objective information regarding intestinal viability, have potential for clinical use, but almost all require the development of robust methodology for intraoperative use and extensive clinical trials.^6^^–^^10^

When searching for new solutions for the intraoperative determination of the intestinal wall condition, it is advisable to consider mesenteric blood flow as an object of study, the disturbances of which are directly related to pathological changes in the intestinal wall. Promising in this direction is the use of a method of optical diagnostics such as hyperspectral imaging, which combines the capabilities of digital imaging and the registration of diffuse reflected light at different wavelengths.^11^ The possibility of non-invasive use of this method, as well as the high potential of mathematical processing provided by spectral resolution, allows us to consider hyperspectral imaging as an alternative to already widely used methods, such as magnetic resonance imaging, intraoperative Dopplerography, and X-ray tomography.^12^ In particular, hyperspectral imaging can be effectively used to determine tissue oxygen saturation and blood content and blood content.^13^^–^^16^ Modern circuitry solutions allow ultra-compact realization of hyperspectral measurements, embedding these systems in laparoscopes or smartphone cameras, significantly expanding the areas of use of this method in medicine.^17^ Due to the high spectral resolution of modern hyperspectral systems, it is possible to realize highly accurate classification models based on classical segmentation methods^18^ and advanced neural network approaches.^19^

Modern studies demonstrate a significant potential of hyperspectral systems in the assessment of ischemic tissue damage. Felli et al.^20^ showed that hyperspectral imaging can reliably differentiate among types of liver ischemia using tissue oxygen saturation indices, even in early-stage lesions. It should be noted that the method not only revealed the degree of hypoxia but was also correlated with histological changes, which confirms its diagnostic value. In surgical practice, hyperspectral imaging finds application in monitoring ischemic preconditioning. The study devoted to the assessment of gastric conduit oxygenation during esophagectomy showed that the method allows not only to identify zones of risk of necrosis development, but also to objectively assess the effectiveness of preconditioning, which is important for reducing the incidence of postoperative complications.^21^ One of the promising directions may be the use of hyperspectral imaging in neurosurgery and neurology. An experimental study conducted in rodent models of cerebral ischemia is known, which showed that the method allows detecting ischemic changes as early as 5 min after occlusion and without the need to use contrast agents.^22^ It should also be noted that significant progress has been achieved in the application of hyperspectral systems in the field of differential diagnosis of ischemic and necrotic changes. The combination of the evaluation of tissue saturation parameters and hemoglobin indices has demonstrated an accuracy of up to 94% in the detection of necrotic areas of the small intestine, significantly exceeding the capabilities of traditional imaging methods.^23^ This is particularly important in the context of minimizing invasive diagnostic procedures. Of particular interest are studies on the application of hyperspectral imaging in the diagnosis of acute mesenteric ischemia. In the corresponding work, the authors demonstrated the high sensitivity (92%) and specificity (88%) of the method in detecting perfusion deficits of the small intestine.^24^ The obtained oxygenation parameters were significantly correlated with the severity of ischemic damage, which, as the authors claim, opens prospects for using hyperspectral imaging (HSI) as an objective criterion for assessing the effectiveness of revascularization.

Moreover, the first steps have already been taken to introduce hyperspectral imaging to determine the viability of intestinal tissues, using machine learning (ML) methods to classify normal and necrotic areas of the small intestine,^25^ as well as to refine the edges of necrotic tissue resection during surgery.^26^ Convolutional neural networks have recently been used quite successfully to determine intestinal wall tissue viability, allowing the high accuracy characteristics (>90%).^27^ Also, the high efficiency of using a deep learning model based on a conditional generative adversarial network has been shown, which allows an intraoperative assessment of intestinal perfusion with accuracy greater than 90%.^28^ There is also a fairly successful application of a feedforward neural network with accuracy when using a recurrent neural network with long short-term memory units (LSTM-RNN) in the assessment of acute intestinal ischemia, which can potentially reduce the high mortality rate and morbidity of patients.^29^ However, despite the fairly successful application of the above-described approaches, there are currently few scientific papers confirming the adequacy of this assessment. This makes it relevant to develop such approaches and test them in real patients with intestinal wall ischemia. In addition, the spectra-based classifier alone may not provide sufficient information for clinicians to assess intestinal wall viability. A comprehensive diagnostic approach should also incorporate physiological parameters, such as tissue oxygen saturation, which can reveal critical insights into altered tissue states.^30^

Thus, the aim of this work was to develop a hyperspectral imaging system for intraoperative assessment of intestinal wall tissue viability in laboratory animals, integrating diagnostic maps of tissue oxygenation with ML classification to distinguish reversible from irreversible ischemia. This approach aims to provide a foundation for future clinical applications in improving surgical decision-making for intestinal ischemia.

## 2 Materials and Methods

### 2.1 Hyperspectral Imaging System

A portable imaging system was constructed on the basis of the SpecimIQ hyperspectral pushbroom camera (Specim, Spectral Imaging Ltd., Oulu, Finland) that provides a spectral resolution of 7 nm within the total range of 400 to 1000 nm. A broadband illumination unit is based on the FRI61F50 fiber optic ring illuminator (ThorLabs, Newton, New Jersey, United States) and the OSL2 irradiation source (ThorLabs), providing a uniform distribution of light intensity in the camera focal plane with an average irradiance of $50\;\mathrm{mW}/{\mathrm{cm}}^{2}$ in the camera field of view. Utilizing the ring illuminator enabled the merging of the illumination and detection axes. The coaxial alignment of the camera’s entrance pupil and the ring light ensured uniform perpendicular illumination of the measurement area. The system was mounted on a reinforced tripod, and the animal was positioned under the camera lens at a distance of 20 cm.

### 2.2 Experimental Methodology

The experiment was carried out on six Wistar laboratory rats (males). The animals were 3 months old, and the mean weight was 194±6  g. The work was performed in accordance with the rules of Good Laboratory Practice (GLP). All manipulations performed were approved by the ethics committee of the Orel Regional Clinical Hospital, developed in accordance with the principles of good laboratory practice GLP (Protocol No. 2 of 18.09.23). The animals were kept in quarantine conditions controlled for temperature, humidity, and cleanliness. A uniform study protocol was developed and used for all animals. A model of intestinal ischemia caused by the formation of ligatures and, as a result, impaired mesenteric blood flow was considered. Before surgery, the animals were kept in isolation for 3 weeks. Surgical intervention was performed using inhalation anesthesia based on 1.5% isoflurane in standard doses. All surgical interventions were performed under strictly aseptic conditions.

After applying anesthesia, the animal was placed on a special fixing platform, and the surgical field was prepared by shaving a section of the anterior abdominal wall and treating it with antiseptic solutions. A midline laparotomy was used as the operative access. The small intestine was carefully exteriorized from the abdominal cavity. The primary vascular supply to the intestine was ligated using 3-0 Capron sutures. Following ligation, the intestine was returned to the abdominal cavity. The laparotomy incision was closed in a continuous fashion with 2-0 polypropylene suture.

To determine the boundary states of the intestine and its pathological changes in the modeling of ischemia, a morphological study was performed at different time intervals after ligature application: after 1, 6, and 12 h. Each animal was referred for laparotomy after a suitable period of time. The intestines were extracted from the abdominal cavity, and an intraoperative evaluation of intestinal viability was performed.

As a result of experimental studies, hyperspectral image arrays of the small intestine were obtained for each time interval of ischemia. To standardize the measured data, we recorded spectra from a diffuse reflectance standard for each measurement. The diffuse reflection standard is a plate made of fluoroplastic F4 with a reflectance of ∼98%.

After visual determination of external symptoms of ischemia and necrosis of the intestinal wall, small intestine resection was performed with subsequent fixation in 10% buffered formalin for 24 h. Representative sections of the intestine were excised and subsequently histologically processed according to standard techniques (using the Milestone Medical LOGOS histological processor), and paraffin blocks were made. Sections 4  μm thick were obtained on a Leica RM2125 RTS rotary microtome (Leica Biosystems, Nussloch, Germany) and stained with hematoxylin and eosin. Pathomorphological changes in the intestinal wall were evaluated under conditions of simulated small intestinal ischemia of different durations. The severity of the morphological picture of ischemia was evaluated according to the Park/Chiu classification.^31^ The key morphological features in the study of acute mesenteric ischemia were considered to be subepithelial edema of the villi, disruption of villus architecture, epithelial desquamation, loss of villi, reactive or destructive changes in the crypts, and, at later stages, transmucosal or transmural infarction. The studies were completed by withdrawing the animals from the experiment according to the protocol approved by the ethics committee.

### 2.3 Pre-Processing of Hyperspectral Data

As pre-processing, normalization to a diffuse reflectance reference and background removal from the images were performed for all measured hyperspectral arrays.

The normalization of the hyperspectral cube obtained to the diffuse reflectance standard, taking into account the dark background correction, was carried out using the following equation

$$I_{\mathrm{ref}}=\frac{I_{\text{raw}}-I_{\text{dark}}}{I_{\text{white}}-I_{\text{dark}}},$$ (1)

where $I_{\text{raw}}$ is the diffuse-reflected light from the measured object, $I_{\text{dark}}$ is the dark current, and $I_{\text{white}}$ is the diffuse-reflected light from the diffuse reflection standard. This approach also mitigates the additive error caused by external light exposure.

The non-informative background was removed by comparing the intensity with the threshold value. According to the results of spectral measurements, the largest differences in the weighted average intensity of pixels in the intestinal wall region compared with the background are observed at a wavelength of 598 nm. As a control value to preserve the informative part, the threshold value was taken as 0.1 for the normalized hyperspectral cube.

### 2.4 Tissue Saturation Calculation

There are already known attempts to determine tissue saturation and hemoglobin oxygen saturation intraoperatively, both on the basis of imaging analysis, using simple two-wave approaches,^32^ and more complex algorithms associated with Taylor series decomposition, based on spectroscopic analysis.^33^

In this work, the tissue saturation parameter was calculated based on the different light absorption of the unbound and oxygen-bound forms of hemoglobin, using the two-wave approach for the near-infrared region based on measured diffuse reflectance coefficients^34^^–^^36^

$$S_{t}O_{2}=\frac{\mu_{\mathrm{Hb}}(750)-\mu_{\mathrm{Hb}}(795)\frac{R_{795}}{R_{750}}}{\mu_{\mathrm{Hb}}(750)-\mu_{{\mathrm{HbO}}_{2}}(750)},$$ (2)

where $R_{795}$ and $R_{750}$ are the measured diffuse reflectance coefficients at wavelengths of 795 and 750 nm, respectively; $\mu_{\mathrm{Hb}}(750)$ and $\mu_{{\mathrm{HbO}}_{2}}(750)$ are the absorption coefficients at the non-isobestic point of deoxygenated and oxygenated blood, respectively; $\mu_{\mathrm{Hb}}(795)$ is the absorption coefficient of deoxygenated blood at the isobestic point.

The calculation using the above equation is applied for each pixel of the recorded hyperspectral array, resulting in diagnostic maps containing information about the level of tissue saturation at each point of the image.

### 2.5 Machine Learning Model

**Fig. 1:**
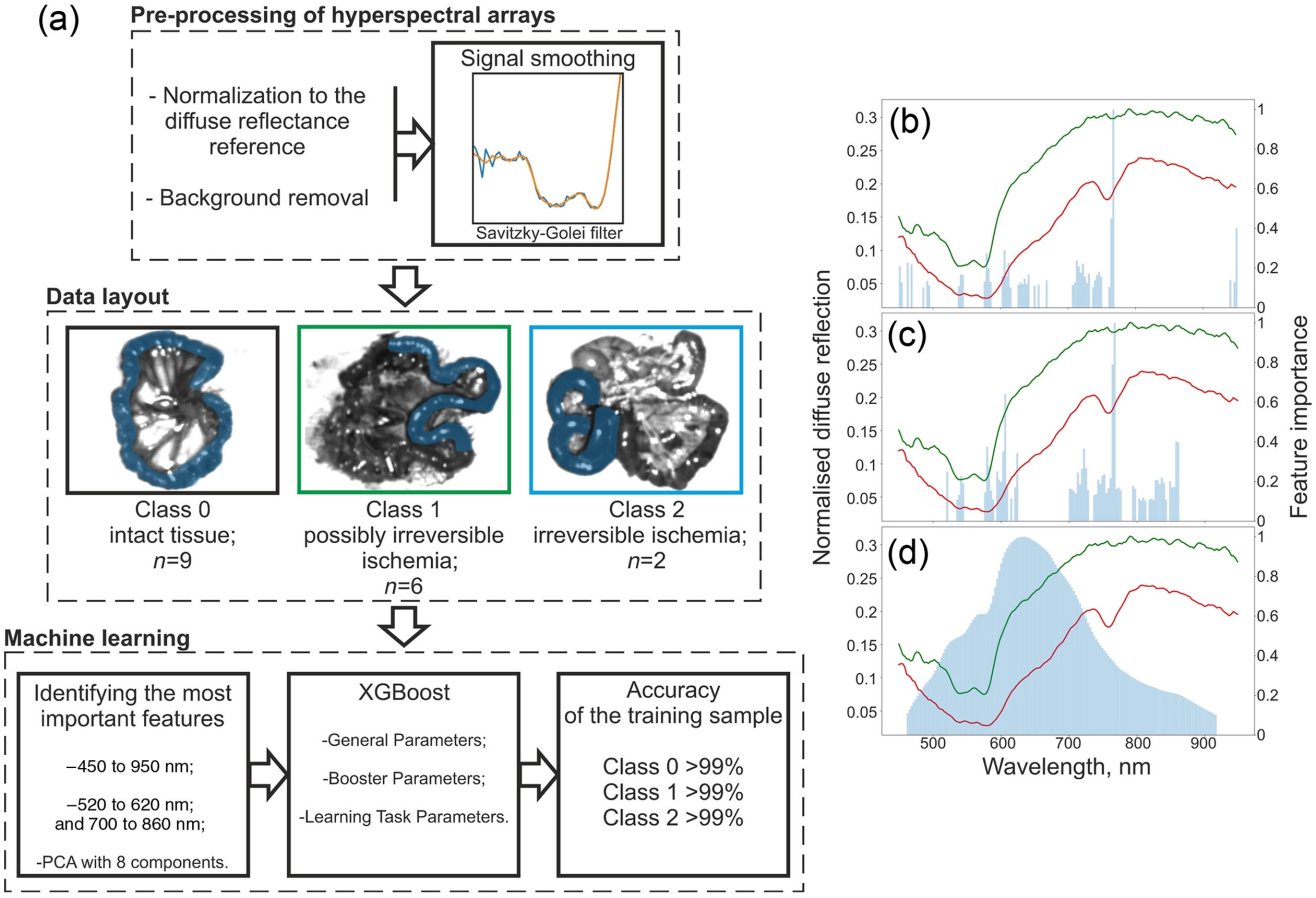
(a) Block diagram of the main steps of ML. Values of normalized weighting coefficients for (b) 450 to 950 nm range, (c) 520 to 620 nm and 700 to 860 nm range. (d) First component of PCA.

The main steps in the application of ML, including pre-processing of hyperspectral arrays, data layout, and ML method, are shown in Fig. 1.

Through continuous development, the application of ML and deep learning techniques has made significant advances in image analysis and recognition, which has also encouraged some researchers to use this field in conjunction with hyperspectral medical images.^37^ In our work, we propose the following ML algorithm described below.

After pre-processing of hyperspectral arrays, to reduce the influence of noise and artifacts on the measured signal, a smoothing method, namely, the Savitzky–Golay filter, was applied.^38^^,^^39^ This method preserves the underlying trends in the data while minimizing the impact of noise components. A second-degree Savitzky–Golay filter (window size = 7) was applied for the normalized hyperspectral cube. The filter parameters were determined experimentally, taking into account the camera’s spectral resolution, the nature of the tissue spectra, and the performance metrics of the classification model.

The data were then labeled according to the Park/Chiu histological classification. Three classes were distinguished: intact tissue, possibly reversible ischemia (grades 1 to 5), and irreversible ischemia (grades 6 to 8). According to histology results, the processed hyperspectral cubes were grouped according to the grades. Then, in each group, for each hyperspectral cube, the area corresponding to a particular grade was highlighted by applying a polygon mask in the spatial coordinates. The selection was performed jointly/supervised by an experienced surgeon-physician who performed local ischemia creation. Next, spectral data were extracted from the selected spatial region for each pixel. In this way, a training sample was created for each class. As the resolution of the recorded hyperspectral cube is 262,144 spectral points, ∼3000 to 7000 spectral points fall on the analyzed areas of the intestine, depending on the occupied image area of each specific intestine area. At the same time, each analyzed spectral point is somewhat different from the other and has a variance, which is justified by the complexity of the organization of human biological tissue. Segmentation results in a spectral dataset for intact tissue (45,377 points, 9 animals in the training set), possibly reversible ischemia (32,715 points, 6 animals in the training set), and irreversible ischemia (11,764 points, 2 animals in the training set).

Further, to assess the importance of different spectral data in the three approaches to feature engineering were used to evaluate the importance of different spectral data in model performance. The first approach included analyzing the data over the entire hyperspectral camera range (450 to 950 nm), except for a 50-nm width at the beginning and end of the range where noise and edge effects can occur, which was also noted in this work,^26^ and results are shown in Fig. 1(b). The second approach was to analyze the data in the characteristic blood absorption regions 520 to 620 nm and 700 to 860 nm, as was also done in this work,^25^ and results are shown in Fig. 1(c). The third approach considered the application of the principal component analysis (PCA) method. This method allows to select the most significant features, as well as to reduce the dimensionality of the features, which is important, especially for tasks with high execution speed. The explained variance method was used to optimally select the number of components, with the goal of reaching a threshold of 99% explained variance. This retained most of the information while reducing the dimensionality to seven components. Only the first component was considered in analyzing the importance of the traits because its proportion of explained variance is 88%. To estimate the weighting coefficients of the principal component, scaling and subsequent downward ranking were also performed. Only those features that contributed 90% of the total contribution to the principal component were considered, and the results of this estimation are presented in Fig. 1(d).

To determine the importance of attributes, their weighting coefficients were used. To normalize the weighting coefficients, all feature values were scaled in an interval from 0 to 1, where 1 corresponds to the most important feature and 0 to the least important. The traits were then ranked in descending order of contribution and retained only attributes contributing ≥90% of the cumulative weight.

According to the estimation results for the first and second approaches, we can observe an uneven contribution of chaotically distributed features, which cannot be used for further model training, as it complicates data interpretation, making it difficult to identify relevant patterns and leading to incorrect model training. PCA revealed that the most informative wavelengths cluster within a single spectral band and vary smoothly across samples. Consequently, all subsequent models were trained on the seven-component PCA representation.

The XGBoost algorithm^40^ was employed for supervised classification. XGBoost was chosen for its combination of high predictive accuracy, computational efficiency, and scalability, which enables near-real-time inference in large datasets. An extensive hyper-parameter search was carried out across the general, booster, and learning task categories. The grid included the learning rate, number of boosting rounds, maximum tree depth, minimum child weight, minimum loss reduction required to create a split, row-wise and column-wise subsampling ratios, and the L1 regularization coefficient. Optimal values were identified via k-fold cross-validation, with the final number of boosting rounds selected using early stopping on the validation folds.

### 2.6 Evaluation Metrics

To assess the quality of the models, we used four accuracy measures: accuracy, the area under the curve (AUC), sensitivity, and specificity. Accuracy is the percentage of correctly classified sections of intestinal wall tissue to the total number of sections examined. AUC in the form of one-versus-all is the area under the receiver operating characteristic (ROC) curve, taking into account the differences of each class from all others and further averaging the results across all classes for an overall performance estimate. This approach allows the classifier performance for each class to be considered separately. Sensitivity is the percentage of true positives in relation to all ischemic intestinal tissues. Specificity was measured by the ability of the classifier to detect true negatives.

## 3 Results and Discussion

### 3.1 Tissue Saturation Analysis

**Fig. 2:**
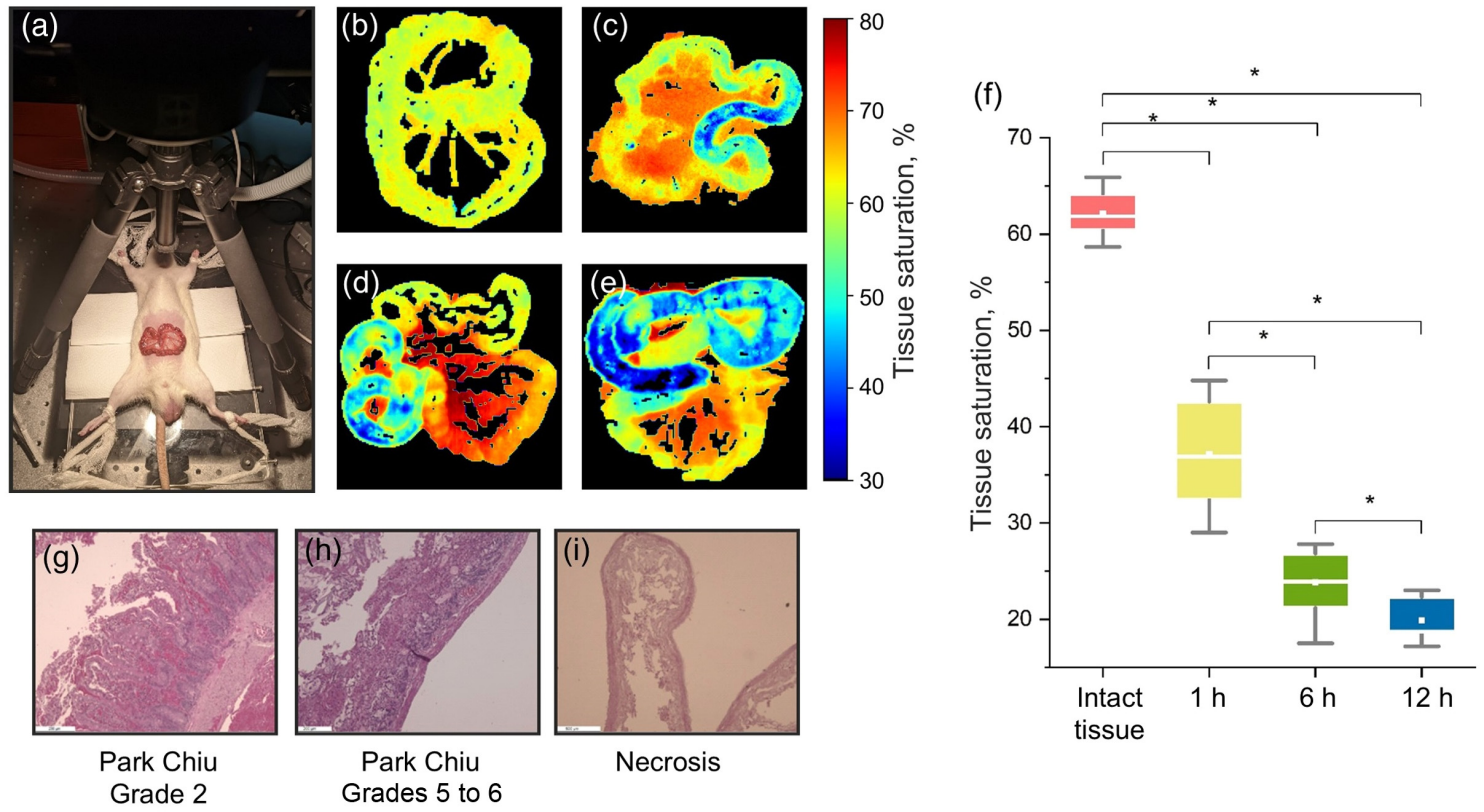
(a) Photograph of an experiment with modeling intestinal wall ischemia in a laboratory animal. Two-dimensional maps of tissue saturation during ischemia: (b) intact intestinal tissue, (c) 1 h after ischemia, (d) 6 h after ischemia, and (e) 12 h after ischemia. (f) Boxplots with tissue saturation values from the corresponding intestinal regions, obtained from two-dimensional maps with a statistical significance level (Mann–Whitney U test) of p<0.05. Corresponding results of histologic analysis for (g) 1 h after ischemia, (h) 6 h after ischemia, and (i) 12 h after ischemia.

Figure 2 shows the results of tissue oxygen saturation measurements during experimentally induced intestinal wall ischemia at 1, 6, and 12 h. The tissue saturation maps, calculated for all stages of simulated intestinal ischemia, visually showed a change in color contrast in the area of intestinal wall ischemia compared with intact areas [Figs. 2(b)–2(e)]. A clear decline in tissue saturation is visible over time, with a progressive shift in color from yellow-red to green-blue in ischemic regions. In intact tissue [Fig. 2(b)], the average saturation was 66±2%. One hour after ligature application [Fig. 2(c)], the saturation decreased to 42±5%. At 6 h [Fig. 2(d)], it dropped further to 26±3%, and by 12 h [Fig. 2(e)], it reached 21±3%. Figure 2(f) presents a boxplot summarizing tissue saturation values across all experimental groups. Statistically significant differences were observed between each time point and the control (intact tissue), as well as among consecutive ischemic stages (Mann–Whitney U test, p<0.05; marked by asterisks). Figures 2(g)–2(i) show histological validation of ischemic damage progression from mild ischemic changes necrosis.

According to the results of the morphological study, statistically significant changes in terms of intestinal wall viability were present at the time of application of the ligature after 1, 6, and 12 h. One hour after modeling ischemia, morphological examination revealed focal desquamation of the epithelium and pronounced villi edema in intestinal fragments. There was moderate lymphohistiocytic infiltration in the intrinsic lamina, edema, and injected vessels in the submucosa. The described changes corresponded to grade 2 intestinal ischemia according to Park/Chiu classification, and the results of histologic analysis are shown in Fig. 2(f). Morphological changes in the intestinal wall 6 h after ischemia modeling were represented by diffuse coagulation necrosis of the villi with hemorrhagic component and focal destruction of the crypts. The submucosa had signs of marked edema, with polymorphocellular infiltration and dilated vessels with blood stasis. The described changes corresponded to grade 5 to 6 intestinal ischemia according to the Park/Chiu classification, and the results of the histological analysis are shown in Fig. 2(g). Twelve hours from the time of the ischemia was created, the intestinal wall was represented by necrotized tissue with an admixture of blood, and the results of histological analysis are shown in Fig. 2(h).

### 3.2 Machine Learning Application

The purpose of applying ML was to classify intestinal wall tissue according to the reversibility of ischemia. Based on the results obtained, we found that possible irreversible ischemia occurs in the histologic classification of Park/Chiu at grades 1 to 5, and irreversible ischemia occurs at grades 6 to 8. The calculated accuracy measures for the three approaches for choosing the downscaling of the original data dimensionality are listed in Table 1. These measures of accuracy were obtained on a test sample (one laboratory animal for possibly reversible ischemia and one laboratory animal for irreversible ischemia). As can be seen from the results obtained, all approaches give high accuracy estimates, but the method using PCA is the most preferred, which showed the following accuracy characteristics: for class 0 (intact tissue), accuracy is 0.98, sensitivity is 0.98, and specificity is 0.94; for class 1 (possibly reversible ischemia), accuracy is 0.95, sensitivity is 0.95, and specificity is 0.96; and for class 2 (irreversible ischemia), accuracy is 0.97, sensitivity is 0.97, and specificity is 0.97, respectively. Thus, in accordance with the accuracy analysis presented in Table 1, the PCA method was chosen as the method for identifying the most important features of the model.

**Table 1:** Accuracy measures of the three approaches for choosing down-dimensionalization of the original data.

	450 to 950 nm	520 to 620 and 700 to 860 nm	PCA
Intact tissue	Acc: 0.97	Acc: 0.97	Acc: 0.98
	AUC: 0.98	AUC: 0.97	AUC: 0.99
	Se: 0.97	Se: 0.97	Se: 0.98
	Sp: 0.85	Sp: 0.78	Sp: 0.94
Possible reversible ischemia	Acc: 0.86	Acc: 0.77	Acc: 0.95
	AUC: 0.98	AUC: 0.97	AUC: 0.99
	Se: 0.86	Se: 0.77	Se: 0.95
	Sp: 0.91	Sp: 0.86	Sp: 0.96
Irreversible ischemia	Acc: 0.92	Acc: 0.90	Acc: 0.97
	AUC: 0.99	AUC: 0.99	AUC: 0.99
	Se: 0.92	Se: 0.90	Se: 0.97
	Sp: 0.92	Sp: 0.88	Sp: 0.97

**Fig. 3:**
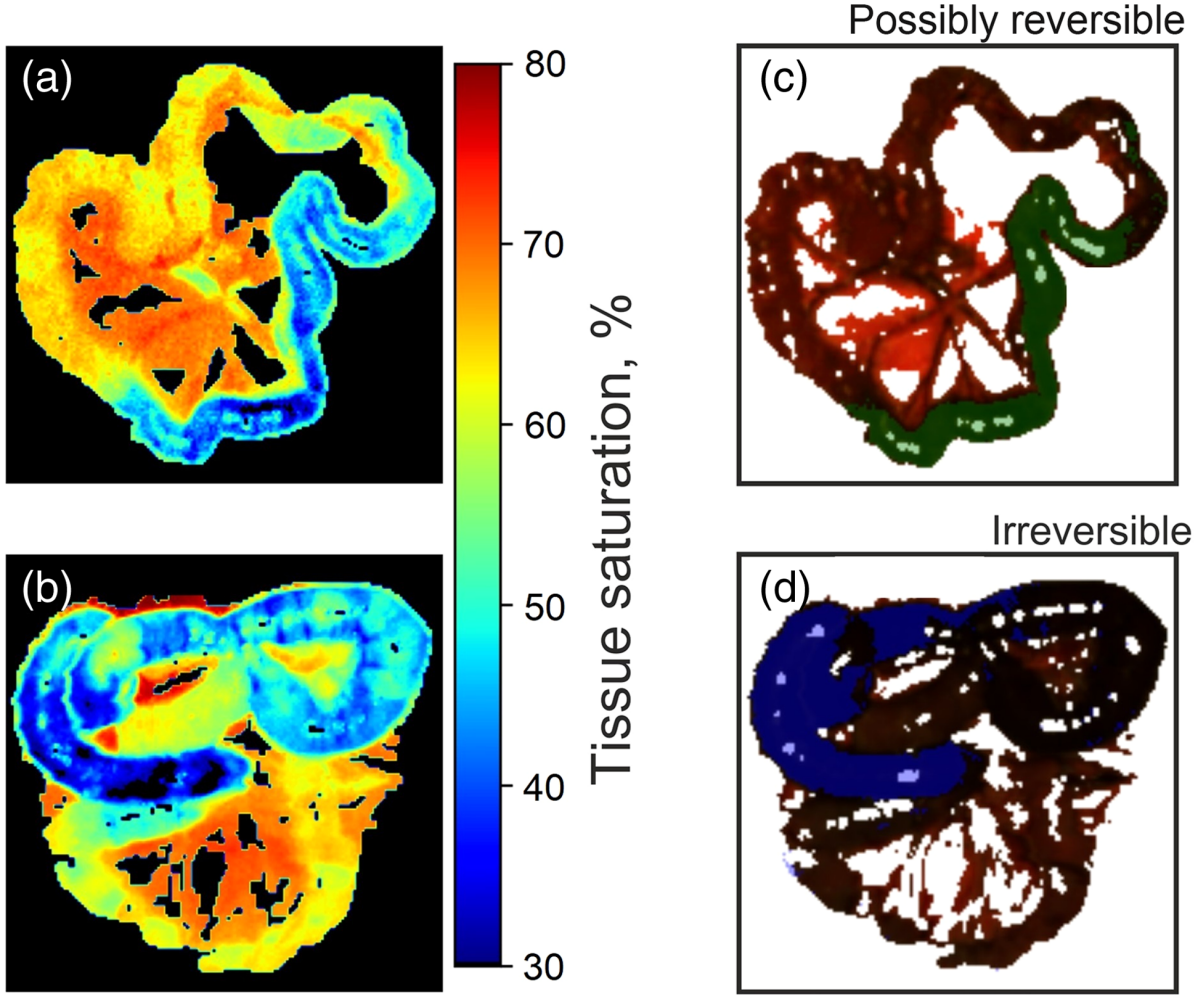
Two-dimensional tissue saturation maps for (a) possibly reversible ischemia and (b) irreversible ischemia. Classification maps for (c) possibly reversible ischemia and (d) irreversible ischemia.

Next, following the developed ML algorithm, classification masks overlayed on the measured bowel images were created and compared with tissue saturation maps for possibly reversible ischemia, shown in Figs. 3(a) and 3(c) and non-reversible ischemia, shown in Figs. 3(b) and 3(d). The classification masks were constructed with a class membership threshold >99%. The results show a visual similarity between the saturation maps and the classification maps, which confirms the accuracy of the application of ML. Moreover, the use of classification maps as a definition of the resection edge appears to be more adequate than tissue saturation maps. As can be seen from the results on the saturation map in Fig. 3(c), the area of ischemia occupies a large part of the intestine loop, shown in blue pseudo-color, and has different levels of saturation over its area. However, on the classification map in Fig. 3(d), only the left part of this intestine loop, which has a darker blue pseudo-color and a saturation value corresponding to irreversible ischemia, is classified as irreversible ischemia, in contrast to the right part of the loop with a tissue saturation value more suitable for the class of possibly irreversible ischemia.

It is important to note that the conservative threshold was chosen to mitigate the influence of inter-animal variability and noise by displaying only the most confident predictions. For any future clinical application of this method, a rigorous, data-driven threshold analysis of a large, independent cohort, accounting for all sources of variability, would be absolutely essential. Furthermore, the pixel-based data split used in this proof-of-concept study risks overestimating performance, as the model could learn animal-specific features. The high metrics primarily demonstrate the method’s feasibility in distinguishing spectral patterns of ischemia. Robust, generalized performance must be validated in future work using a leave-one-out cross-validation strategy to ensure the model generalizes to new subjects.

### 3.3 Pilot Clinical Report

**Fig. 4:**
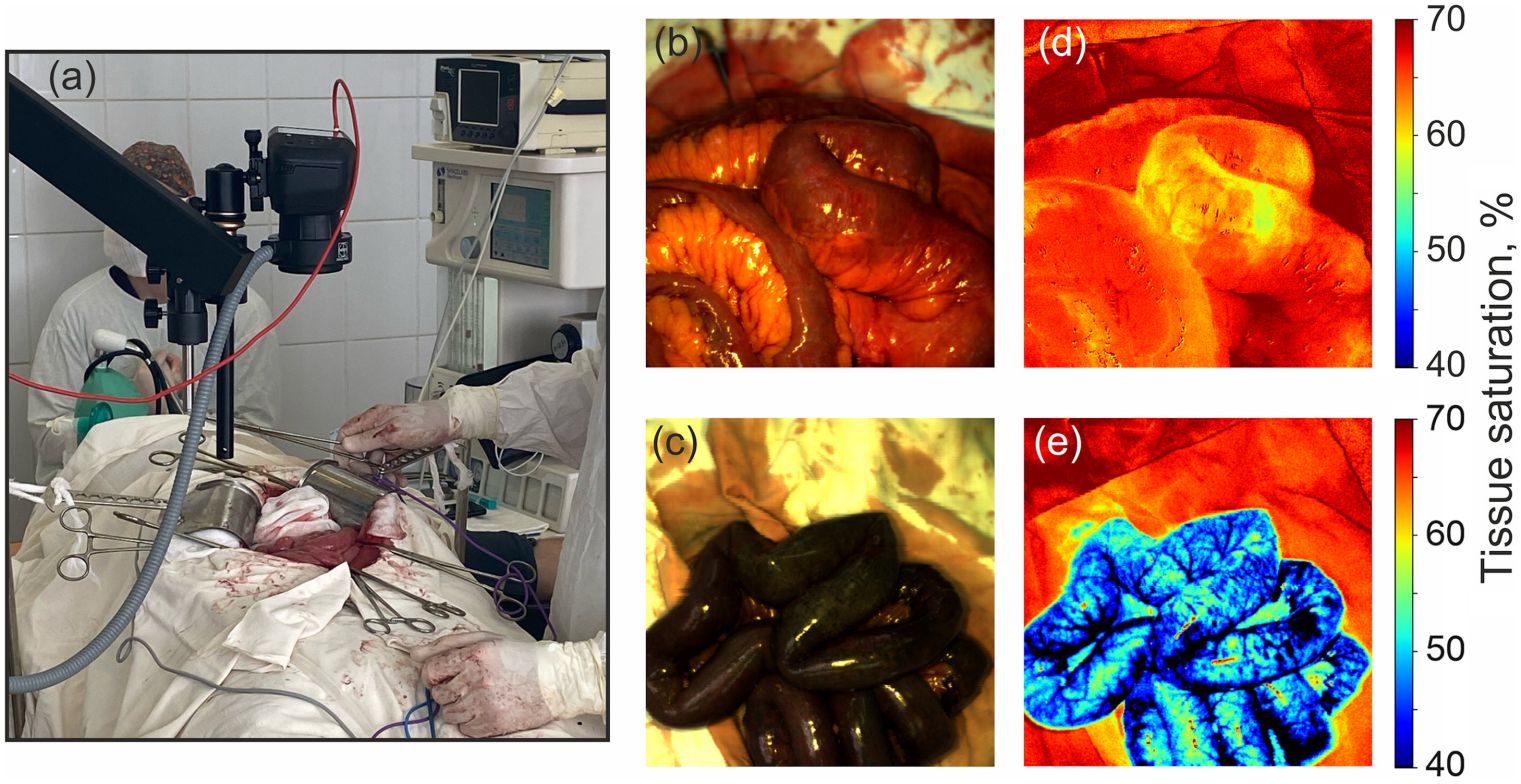
(a) Photograph of the system being used during a surgical operation. (b) Photograph of the intestine with infringement. (d) Two-dimensional tissue saturation maps of the intestinal with infringement. (c) Photograph of the intestine with total necrosis. (e) Two-dimensional tissue saturation maps of the intestinal with total necrosis.

We applied the hyperspectral imaging system presented in this paper to determine the viability of the intestinal wall of patients at the Orel Regional Clinical Hospital. For use in a surgical room, we placed the developed system on a medical stand, the camera together with the ring emitter was fixed on a reinforced tripod, thus providing convenient aiming of the camera lens by the doctor at the investigated intestinal area. The Fig. 4(a) shows a photograph of the use of our system during a surgical procedure. As can be seen from the photo, the system does not interfere with the surgeon’s ability to perform surgery due to its overall dimensions and positioning, but can be quickly assembled with a movable reinforced tripod if the surgeon needs free space. All the manipulations performed were approved by the ethics Committee of the Orel Regional Hospital (Orel, Russia), developed in accordance with the principles of GLP (Protocol No. 2 dated 09/18/13).

The study included two patients, each of whom underwent diagnostic laparotomy followed by removal of intestinal obstruction. The first patient was a 60-year-old woman diagnosed with acute adhesive bowel obstruction; a photograph of the isolated intestine and a tissue saturation map are shown in Fig. 4(b). The tissue saturation map of the intestine clearly shows an area of reduced saturation corresponding to the pinched area; it is identified in the image as the upper wrapped loop of the intestine. The average values of tissue saturation in the pinched area are 60% compared with the unchanged area with average values of 65%. The second patient was a 74-year-old woman diagnosed with acute segment I mesenteric thrombosis with necrosis of the small intestine and the right half of the colon; a photograph of the isolated intestine and a tissue saturation map are shown in Fig. 4(c). On the saturation map, the entire intestinal wall is necrotic tissue lesions, with average tissue saturation values of 40%. The arcade vessels of the intestine are particularly prominent, with very low saturation values and a corresponding dark blue pseudo-color.

Based on the results, we can conclude that the system is sensitive to detecting changes in the tissue saturation parameter, both for the example of possibly reversible ischemia and for irreversible ischemia. Preliminarily, the threshold of tissue saturation values for irreversible ischemia is higher in patients than in model animals. The tissue saturation values for possibly reversible ischemia are generally also higher in patients than in model animals. Machine learning was not applied to these results because it is trained only on a dataset of laboratory animals and cannot be adequately applied to the classification of human intestinal tissue.

In future research, the development of machine learning models specifically trained on human datasets will be a critical focus. This will involve collecting and annotating hyperspectral imaging data from human patients with intestinal ischemia to create a robust and representative training set. Advanced techniques such as transfer learning may be employed to adapt existing animal-trained models to human data, whereas convolutional neural networks and other deep learning architectures will be explored to improve classification accuracy and generalizability. In addition, clustering algorithms will be integrated to better identify and segment specific areas of ischemic tissue in human intestines.

It is worth noting here that the key limitations of the use of machine learning may be related to the specifics of clinical cases: patients admitted to abdominal surgery often present with comorbidities that can themselves cause ischemic changes in the intestinal wall and complicate the interpretation of spectral data. An additional challenge is class labeling, as histological verification is primarily possible only during resection, when a segment of the intestine is already in a state of irreversible ischemia, making it difficult to confirm intermediate stages of damage. To mitigate these limitations, future research plans include the use of transfer learning strategies, regularization methods, and the integration of data from multiple modalities. This approach is expected to enhance the robustness of deep learning models and reduce the impact of clinical heterogeneity and incomplete labeling.

## 4 Conclusion

Our study showed that the use of hyperspectral imaging can provide information on the state of microcirculation in the intestinal wall by assessing tissue saturation. Additional application of machine learning techniques can refine the area of ischemic changes, giving more accurate information about the reversibility of ischemia and possibly the resection margin of intestinal tissue. The results obtained serve as a basis for the application of hyperspectral imaging in intraoperative diagnostics of the intestinal condition associated with impaired blood supply (pinched hernia, intestinal twist, strangulation intestinal obstruction, etc.) along with the visual method of Kerte. The presented machine learning algorithm really makes it possible to specify the area of ischemic changes, informing the doctor about the reversibility of ischemia and the edge of intestinal tissue resection with an accuracy of at least 95%.

The use of hyperspectral imaging is reasonable as a method of non-invasive and objective intraoperative assessment of ischemic damage to the intestinal wall without additional use of contrast agents, allowing to get an idea of intestinal microcirculation and a number of quantitative diagnostic tissue parameters. The system is designed for seamless integration into the surgical workflow; it is mounted on a mobile stand that does not obstruct the operative field, and the computational analysis provides classification results in real-time on a standard laptop without requiring specialized GPU servers. However, we explicitly acknowledge that a significant limitation is the current scope of our clinical data. The translation of this technology into routine practice is objectively a medium-term prospect, contingent upon successful completion of extensive, multi-center clinical studies to rigorously validate the algorithm’s performance across a wider and more diverse patient population. Furthermore, securing the necessary regulatory approvals will be an essential step prior to clinical adoption.

## Data Availability

The data underlying the results may be obtained from the authors upon reasonable request.
